# Strong, ductile, and thermally stable Cu-based metal-intermetallic nanostructured composites

**DOI:** 10.1038/srep40409

**Published:** 2017-01-09

**Authors:** Keith J. Dusoe, Sriram Vijayan, Thomas R. Bissell, Jie Chen, Jack E. Morley, Leopolodo Valencia, Avinash M. Dongare, Mark Aindow, Seok-Woo Lee

**Affiliations:** 1 Department of Materials Science and Engineering & Institute of Materials Science, 97 North Eagleville Road, Unit 3136, Storrs, CT, 06269-3136, USA

## Abstract

Bulk metallic glasses (BMGs) and nanocrystalline metals (NMs) have been extensively investigated due to their superior strengths and elastic limits. Despite these excellent mechanical properties, low ductility at room temperature and poor microstructural stability at elevated temperatures often limit their practical applications. Thus, there is a need for a metallic material system that can overcome these performance limits of BMGs and NMs. Here, we present novel Cu-based metal-intermetallic nanostructured composites (MINCs), which exhibit high ultimate compressive strengths (over 2 GPa), high compressive failure strain (over 20%), and superior microstructural stability even at temperatures above the glass transition temperature of Cu-based BMGs. Rapid solidification produces a unique ultra-fine microstructure that contains a large volume fraction of Cu_5_Zr superlattice intermetallic compound; this contributes to the high strength and superior thermal stability. Mechanical and microstructural characterizations reveal that substantial accumulation of phase boundary sliding at metal/intermetallic interfaces accounts for the extensive ductility observed.

Despite the excellent ductility that is characteristic of most pure metals, their application as structural materials has been limited due to their poor specific strength^1^. Various strengthening methods have been used in an attempt to improve the specific strength of metals^2^. Most notable in pure metals, the reduction of grain size to the nanometer scale has been carried out to produce ultra-high strength metals, which have been termed nanocrystalline (NC) metals^3^,^4^,^5^,^6^,^7^. Reduction of the grain size in a multi-component alloy beyond the nanometer regime can lead to amorphization, resulting in a bulk metallic glass (BMG) which has been regarded as one of the strongest metallic systems^8^,^9^,^10^,^11^,^12^,^13^. Although these processes are successful in increasing the strengths of metallic systems, they generally result in an increase in brittleness. For instance, Co-based BMGs exhibit yield strengths of up to 5 GPa, which is even higher than that of ceramic alumina (2.5 GPa), but exhibit negligible plasticity at room temperature^14^. NC metals also generally exhibit poor ductility at room temperature because of limitations on dislocation motion in the nano-sized grains. It is only under particular circumstances, such as the introduction of nano-spaced twin boundaries or abnormally large grains, that enhanced ductility can be exhibited in NC metals^15^,^16^. The most challenging issue for both BMGs and NC metals is the lack of high temperature microstructural stability^6^,^9^. The glass transition of BMGs allows for various plastic forming methods to be available at relatively low temperatures. However, this formability implies poor high temperature mechanical properties since BMGs soften dramatically above their glass transition temperatures^17^,^18^,^19^. A similar challenge is present at high temperatures for NC metals because of the thermodynamic favorability for grain growth to occur. Although the ductility of BMGs and NC metals has been improved by the introduction of a ductile second phase, the thermal stability of these composites would be still poor since the matrix is a BMG or NC metal. Thus, the identification of a new material system that possesses high strength, high ductility as well as superior microstructural stability at an elevated temperature has long been regarded as a critical objective in the development of advanced structural materials.

In this work, we present novel ternary Cu-based metal-intermetallic nanostructured composites (MINCs), which successfully combine high strength, high ductility and superior microstructural stability at an elevated temperature. We carefully selected Cu-Zr-Ti and Cu-Zr-Al ternary systems following three major considerations. First, the number of alloying components was intentionally chosen to be less than four elements because it allows for consultation of Gibbs phase diagrams and the scientific study of the phase formation mechanisms. Second, in selecting the two major alloying components, Cu-Zr compositions located at the narrow eutectic region between ductile metal (Cu) and strong intermetallic compounds were considered (The three possible phase diagrams are available in Supplementary Information)^20^,^21^,^22^. This narrow compositional window in the eutectic region allows us to achieve a composite having a large volume fraction (~60%) of intermetallic compounds with minimal use of Zr, an expensive secondary alloying element, according to Gibbs phase rule. For comparison, the MINCs in this study make use of less than 11 at.% of Zr, while Cu-Zr containing BMGs typically contain over 30 at.% of Zr. This highlights the economic advantage of our MINCs. Third, the addition of a third element (Ti or Al) was chosen to further enhance the composite by influencing the production of a strong, supersaturated Cu phase or a NC intermetallic phase, respectively, which makes an additional contribution to the high strength of the MINCs^23^,^24^,^25^.

## Results

**Figure 1:**
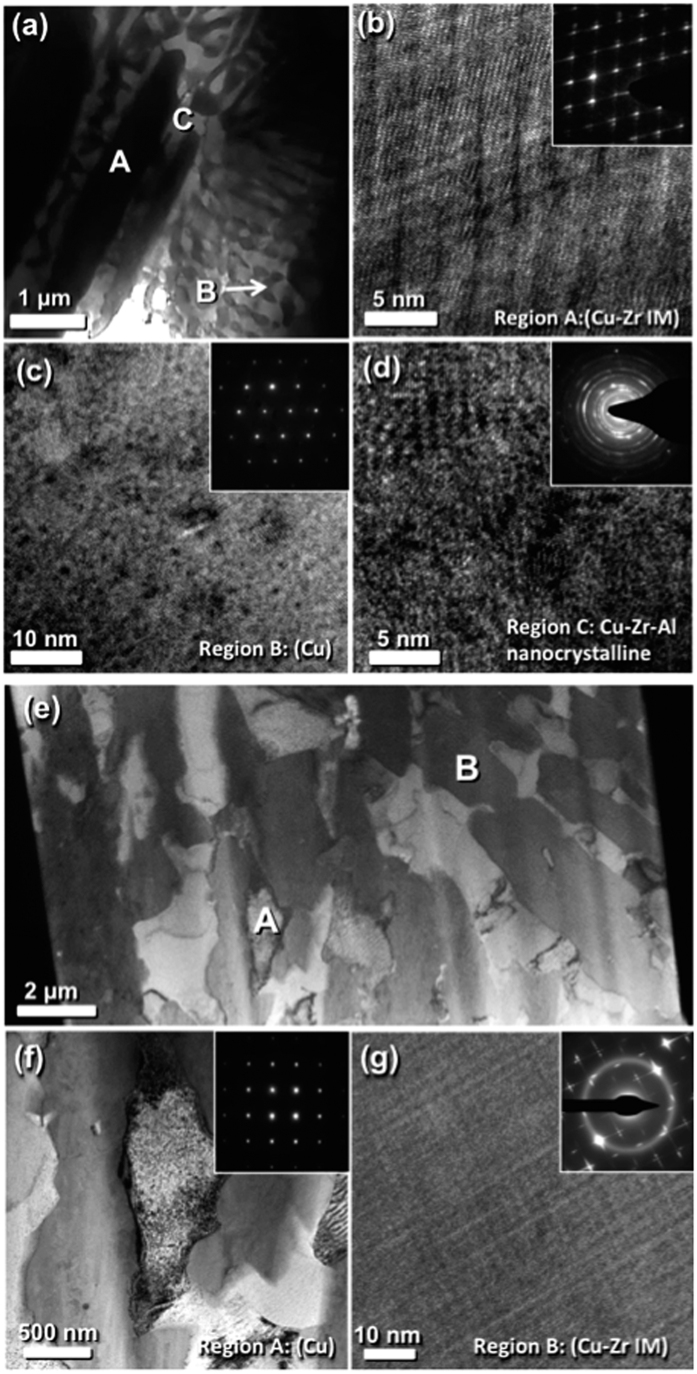
Transmission electron microscope bright field images of (**a**) microstructure, (**b**) Cu-Zr IM phase, (**c**) Cu phase, (**d**) nanocrystalline phase of Cu_86_Zr_11_Al_3_, and of (**e**) microstructure, (**f**) Cu-Zr IM phase and (**g**) Cu phase of Cu_85_Zr_10_Ti_5_. Each inset is the selected area diffraction pattern of the corresponding image. Small black contrast in (**c,f**) would be produced by FIB milling during TEM sample preparation. The fine line structures in (**b,g**) are produced by the alternating layered superlattice structure of Cu_5_Zr.

For the compositions chosen in this study, rapid solidification allows for the generation of unique, ultra-fine metal-intermetallic microstructures. Cylindrical specimens having diameters, D, of 1.5, 2, and 3 mm were fabricated by *in-situ* suction casting into water-chilled molds in an arc melter (See Materials and Methods). Figure 1 shows transmission electron microscope (TEM) bright field (BF) images of specimens with D = 1.5 mm. The Cu_86_Zr_11_Al_3_ system contains three phases (Fig. 1(a)): a Cu-Zr superlattice intermetallic phase (Fig. 1(b)); a bulk Cu phase (Fig. 1(c)) and a Cu-Zr-Al ternary intermetallic phase (*τ* phase, Fig. 1(d)) as predicted by the Gibbs phase diagram. It is interesting to observe that the Cu_85_Zr_10_Ti_5_ system features only two phases (Fig. 1(e)): a Cu phase (Fig. 1(f)) as well as the Cu-Zr superlattice intermetallic phase (Fig. 1(g)). From high-angle annular dark-field (HAADF) analysis (see Supplementary Information), we confirmed that the Cu phase is supersaturated with Ti. Based on the Cu-Ti phase diagram, Ti is soluble in Cu up to 7.5 at.% at 900 °C, but exhibits negligible solubility at room temperature. The rapid solidification process would kinetically trap Ti atoms in the Cu phases, resulting in a non-equilibrium supersaturated phase. We also observed a Cu_4_Ti phase, but its volume fraction is small. So, its contribution to mechanical properties would be negligible. In general, the length scale of each phase ranges from sub-micrometer to a few micrometers. However, the Cu-Zr-Al system contains a nanocrystalline *τ* phase with grain sizes ranging from 5 to 10 nm. The common microstructural feature in both Cu-Zr-Al and Cu-Zr-Ti systems is a large volume fraction of Cu-Zr binary intermetallic compound. Based upon previous studies in the literature, the binary intermetallic compounds that can form in this composition range are: Cu_5_Zr, Cu_51_Zr_14_ and Cu_9_Zr_2_^20^,^21^,^22^. These three phases have been observed under different experimental conditions leading to some debate regarding both their stability and the mechanisms by which they form. The current consensus is that Cu_51_Zr_14_ is a stable phase that exists as a line compound extending up to the liquidus. The high-temperature phase is Cu_5_Zr, which forms as a peritectic product between liquid and Cu_51_Zr_14_ at 1013 °C and decomposes to Cu+Cu_51_Zr_14_ at 591 °C^22^. It is therefore most likely that rapid quenching of Cu-Zr-Ti alloys in these composition ranges retains the high temperature metastable Cu_5_Zr phase with a distorted lattice. This hypothesis is consistent with our analysis of SADPs acquired from the Cu-Zr phase in the as-cast Cu-Zr-Ti alloy as shown in Fig. 1(g). Furthermore, there is evidence for the presence of chemical fluctuations and residual stresses within the intermetallic phase, which complicates the diffraction analysis^26^. In our MINCs, no special growth orientation of intermetallic compound with respect to the casting direction was observed. Rather, all of the intermetallic phases appear to be oriented randomly as confirmed in low magnification images. The large volume fraction (~60%) of the intermetallic phase is nearly entirely bound to the ductile Cu phase, which confines and restricts the plastic deformation of the latter phase. We believe that the unique microstructures of the MINCs presented in this work are directly responsible for the superior mechanical properties and thermal stability that they display.

**Figure 2:**
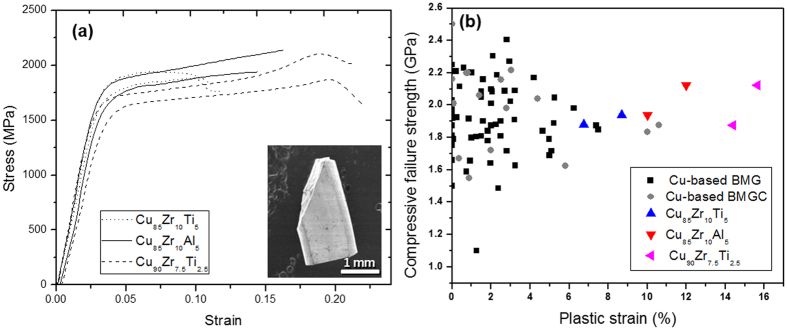
(**a**) Engineering stress-strain curves of Cu-based MINCs in this study. The inset is the scanning electron microscope image of fractured Cu_85_Zr_10_Ti_5_ specimen. (**b**) Plot of plastic strain vs. fracture strength of Cu-based bulk metallic glasses, Cu-based bulk metallic glass composites, and Cu-based MINCs in this study. Note that we obtained data from Cu alloys that contains Cu as the major composition component. Reference of each data point is available in Supplementary Information.

Quasi-static uniaxial compression testing was performed at a constant strain rate of 2.5 × 10^−4^ s^−1^. Figure 2(a) shows the stress-strain data from samples with D = 1.5 mm for two Cu-Zr-Ti compositions and one Cu-Zr-Al composition. Each of these materials exhibited superior strength and ductility. Figure 2(b) is a plot of plastic strain vs. fracture strength for Cu-based BMGs, Cu-based bulk metallic glass composites (BMGCs), and the Cu-based MINCs presented in this work. The reference for each data point is available in the Supplementary Information. Note that our MINCs exhibit high yield strengths (1.5~1.8 GPa) and high fracture strengths (1.7~2.2 GPa), which are similar to those typically exhibited by Cu-based BMGs or BMGCs (~2 GPa). More surprisingly, the amount of plastic strain displayed by our MINCs is much larger than most of the competing materials listed. Particularly notable is Cu_90_Zr_7.5_Ti_2.5_, which shows ~18% plastic strain and ~2.2 GPa ultimate compressive strength at fracture. The inset in Fig. 2(a) shows a Cu_85_Zr_10_Ti_5_ specimen post-fracture, illustrating that shear fracture was the resulting failure mechanism, as typically observed in BMGs or NC metals. All MINCs in this study fractured in the same way, implying that the plasticity and fracture of these MINC result primarily from shear process. The enhanced ductility observed in our MINCs is somewhat unexpected and counterintuitive, since their microstructures include a fairly large volume fraction of intermetallic compounds, which typically are subject to catastrophic, brittle fracture. Image analysis confirmed that the volume fractions of these intermetallic compounds in our MINCs are nearly 60%. Additionally, because the smaller Cu grains are entirely surrounded by the intermetallic compound, dislocation plasticity in these grains is not expected to contribute significantly to the global plasticity of our material. These considerations lead to the assumption that unique non-dislocation-based plasticity mechanisms are available in our MINCs. It is of significant interest to understand the unique shear deformation mechanisms present and active in our material.

The microstructural stability of the MINCs presented in this work was evaluated at an elevated temperature. Intermetallic compounds, such as Ni_3_Al, typically exhibit excellent high temperature stability making them candidates as a material for use in high temperature applications^27^,^28^. The as-cast samples were annealed at 500 °C for 24 h; this temperature exceeds the glass transition temperature of most Cu-based bulk metallic glasses (~430 °C). It was confirmed that the microstructural length scale does not change significantly and that the MINCs exhibit similar superlattice-type electron diffraction patterns before and after annealing. This suggests that the crystal structure of the intermetallic compound remains largely unchanged during annealing and so this phase has good high temperature stability.

## Discussion

### High strength

Our Cu-based MINCs exhibit high strengths, which are comparable to those of BMGs and BMGCs (Fig. 2(b)) as well as other ternary, quaternary, and quinary MINCs (Table 1) fabricated by the similar rapid solidification methods. It is surprising that our Cu-based ternary MINCs with compositions of Cu greater than 80 at.% show superior yield strengths compared to Ti-based and Fe-based quaternary/quinary MINCs produced using similar fabrication process^29^,^30^. The sample that exhibited the highest ultimate compressive strength contains even 90 at.% of Cu. These results are of note because Ti- and Fe-based alloys are generally stronger than Cu-based alloys. Additionally, it was observed that the more rapidly solidified specimens (D = 1.5 mm) are not only stronger but also more ductile than the slowly cooled specimens (D = 3 mm) (See also the stress-strain curves in Supplementary Information). The higher strengths of the more rapidly solidified specimens could be understood well in terms of conventional Hall-Petch type effects, but this would not account for their higher ductilities, which will be discussed in the next section.

There are several factors that could contribute to the high strength displayed by our MINCs. It is noted that the length scales of the microstructures in our MINCs ranges from sub-micrometer to micrometers. The ultra-fine grained microstructure would contribute to the overall strength based on Hall-Petch type strengthening, especially for the ductile Cu phase due to the limited dislocation plasticity in small Cu grains. In the Cu-Zr-Ti system, solid solution strengthening would additionally contribute to the high strength. The Cu phase is supersaturated with Ti, resulting in a phase that would be much stronger than pure Cu. When considering the Cu-Zr-Al system, the presence of a nanocrystalline phase further promotes hardening in that system. However, since the microstructures comprise around 60% by volume of the Cu-Zr binary intermetallic compound phase, we anticipate that the inherent strength of this phase will be the dominant factor in the enhanced strength of our MINCs. In the SADPs obtained from the Cu-Zr binary intermetallic compound, there are satellite spots which imply that the phase exhibits a superlattice structure. In general, it is expected that this complex crystal structure will inhibit slip processes in our MINCs. Furthermore, the strong directional bonding between Cu and Zr atoms in the intermetallic phase produces a chemically and mechanically robust phase. We confirmed that this intermetallic compound was intact after being exposed to a 50% Nital etchant solution for several minutes, while the Cu phase is entirely removed (See Supplementary Information).

We also conducted Density Functional Theory (DFT) calculations to compare the slip resistance and theoretical shear strength between C_5_Zr intermetallic compound and Cu phases. For the minimum energy slip process of the two materials, we found the theoretical strength (~8 GPa) of Cu_5_Zr is much higher than that of the Cu phase (~3 GPa) (See Supplementary Information). Therefore, the large volume fraction (~60%) of a strong intermetallic phase would be the main contributor to the high strengths measured in our MINCs.

### Enhanced ductility

**Figure 3:**
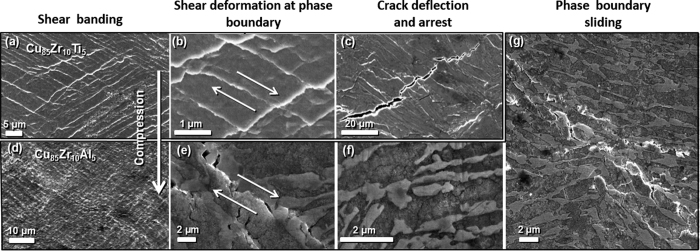
Scanning electron microscope images of (**a**) side surface, (**b**) shear deformation at phase boundary (phase boundary sliding), and (**c**) deflection of microcracks in Cu-Zr IM phase of Cu_85_Zr_10_Ti_5_, and of (**d**) side surface, (**e**) shear deformation at phase boundary, and (**f**) microcrack trapping in Cu-Zr IM phase of Cu_85_Zr_10_Al_5_. (**g**) large-scale shear deformation along phase boundaries (the long-range connection of phase boundary sliding).

In an attempt to fully understand the unexpectedly large ductility in our specimens, we partially polished the side surface (See the inset in Fig. 2(a)), and applied a 30% Nital solution for 5 seconds to selectively etch the sample, allowing for visualization of each phase by scanning electron microscope (SEM). For Cu_85_Zr_10_Ti_5_ and Cu_85_Zr_10_Al_5_, both the polished and fractured surfaces were carefully examined to understand the deformation mechanisms. Two confirmations for the extensive ductility displayed in each MINCs were noted. First, micro-cracks in the brittle intermetallic phase are blunted by the ductile Cu phase (Fig. 3(c,f)). Because the intermetallic phase surrounds a ductile Cu phase, micro-crack propagation does not easily occur as there is crack arrest and propagation path lengthening which occurs at the boundary of these two phases. Second, and most notable, the substantial amount of localized shear processes (sliding) at the phase boundary between the metal and intermetallic phase is observed across the entirety of the specimen volume (Fig. 3(b,c,e,f)). This shear deformation preferentially occurs along phase boundaries orientated at nearly 45°, which implies that plasticity in our MINCs occurs dominantly under the maximum shear stress. The microscopic mechanism of phase boundary sliding would be as follows. During the course of global plastic deformation, ductile Cu phases could be plastically deformed, and Cu_5_Zr phases could be fractured by micro-cracking. These two processes must occur mutually due to strain compatibility, and microscopic plasticity would be transferred through phase boundaries that are oriented nearly 45° from the direction of deformation. Thus, phase boundary sliding results from propagation of plastic deformation in the Cu phase and micro-cracking of the Cu_5_Zr phase along phase boundaries. This unusual deformation mechanism accounts for the extensive ductility exhibited by our MINCs even though the microstructure features a large volume fraction of intermetallic compounds. Figure 3(g) illustrates the connection and joining of sliding between the phase boundaries, which results in global shear plasticity. When local shear deformation propagates completely across the specimen, final shear fracture will occur (the inset of Fig. 2(a)). The ultra-fine microstructure of our rapidly cooled specimens would provide an optimum morphology and connectivity of phase boundaries. The elongated shape of the intermetallic phases, which are oriented 45° from the loading direction, serves as a smooth sliding surface which allows for phase boundary sliding to occur easily and accounts for the extensive plasticity observed. Regions of the intermetallic phase, which are not oriented at 45°, would effectively obstruct the continuous propagation of shear deformation, preventing catastrophic brittle fracture from occurring.

Furthermore, Table 1 shows that the more rapidly solidified specimens of Cu_85_Zr_10_Ti_5_ (D = 1.5 mm) is more ductile than the slowly cooled specimens (D = 3 mm and ingot). Most metals and alloys exhibit larger ductility when they are slowly cooled because slow solidification produces large grains that can exhibit dislocation plasticity. Our counterintuitive results confirm the dependence of ductility upon the morphology of phase boundaries, not dislocation plasticity. The coarser microstructure of slowly cooled specimens does not accommodate the long-range, smooth sliding at phase boundaries (see Fig. S12 in Supplementary Information) and is less ductile than their rapidly cooled counterparts, which feature a fine microstructure. Significant compressive ductility of Cu-based MINCs motivates us to investigate their tensile ductility, which is practically important. Thus, it would be worthwhile to investigate tensile ductility of Cu-based MINCs as a future work.

### Superior microstructural stability at elevated temperatures

**Figure 4:**
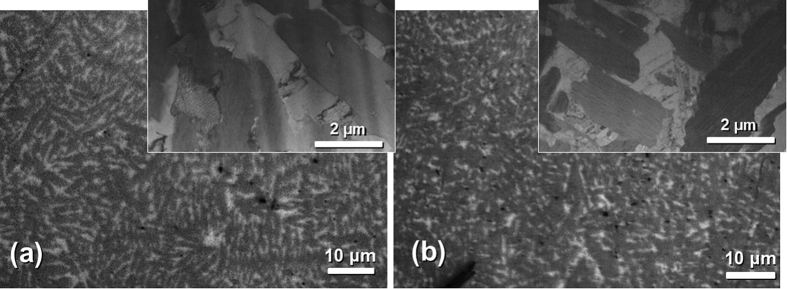
Visible light microscopy images of Cu_85_Zr_10_Ti_5_, with TEM BF image inset, of (**a**) the as-cast sample and (**b**) sample after annealing for 24 hours at 500 °C.

Annealing the as-cast samples at 500 °C for 24 hours confirmed that our Cu-Zr-Ti MINC is thermally stable, even above the glass transition temperatures of many Cu-based bulk metallic glasses (~430 °C). As revealed by TEM analysis, the intermetallic compound formed in our composite has a complex crystal structure and features strong, directional bonding. Grain growth would be significantly limited for this phase since diffusion of each element to their preferred lattice site is unlikely to occur within the time frame investigated in this study. In comparison of the MINCs presented in this work to the high temperature microstructural stability of brass, a Cu-Zn alloy, it is noted that the grain size of brass becomes nearly four times larger after only 15 min of annealing near 500 °C^31^. This implies that grains of conventional Cu alloys will undergo rapid and considerable growth near 500 °C when surrounded by grains of the same composition. The Cu grains in our MINCs are completely bounded by a thermally stable intermetallic compound, which acts as a diffusion barrier to Cu atoms, preventing the rapid coarsening of Cu grains in our material. Under similar annealing conditions, the microstructural length scale of our Cu-Zr-Ti MINC does not change significantly (Fig. 4). The SADPs from the annealed samples reveal that the metastable Cu_5_Zr phase is retained. This is consistent with previous studies on Cu-Zr alloys with such compositions^21^, in which it was shown that the kinetics of the eutectoid transformation from Cu_5_Zr to Cu+Cu_51_Zr_14_ are sluggish. In this previous study, annealing at 500 °C (91 °C below the eutectoid transformation temperature) for 24 hours was not sufficient for the transformation to proceed to completion. Furthermore, the persistence of the length scale for the microstructures in our as-cast and annealed samples implies that our Cu-based MINCs are stable at temperatures higher than the glass transition of Cu-based BMGs. These materials exhibit superior microstructural stability at elevated temperature, as compared to other Cu alloys. Then, it is of interest to study how this excellent thermal stability influences mechanical properties at elevated temperatures under dynamic loading or creep conditions. So, it would be important to investigate high temperature tensile testing as a future research effort.

## Concluding Remarks

For many decades, material scientists have strived to develop a structural material, which combines high strength, high ductility, and superior high temperature stability. Generally, achieving excellence in one of the listed properties comes at the expense of the others. In this work, we have present Cu-based metal-intermetallic nanostructured composites, which exhibit unique microstructures and an un-conventional deformation mechanism (low-temperature phase boundary sliding), resulting in ultra-high strength, high ductility and superior temperature stability when compared to other advanced structural Cu alloys including Cu-based BMGs and BMGCs. The attractive combination of properties exhibited by our MINC materials makes them excellent candidates for a wide variety of structural engineering applications including aerospace, military, automotive, and construction applications.

## Materials and Method

Cu-Zr-Ti and Cu-Zr-Al ingots were prepared by pre-alloying sufficient amounts of elemental pieces of Cu (99.999%), Zr (99.8%), Ti (99.9%) and Al (99.9%) in an arc melter with a water-chilled copper crucible in a high purity Ar atmosphere. Each ingot was re-melted at least three times to ensure that chemical homogeneity was achieved. Cylindrical specimens having diameters, D, of 1.5, 2 and 3 mm were prepared by melting the ingot and suction casting into water-chilled copper molds. Cast cylinders and specimens prepared from the bulk ingot having an aspect ratio of 2.5:1~3:1 were used for quasi-static uniaxial compression testing at room temperature at an initial strain rate of 2.5 × 10^−4^ s^−1^. Phase and compositional analyses were carried out by high-resolution transmission electron microscopy (HRTEM) coupled with energy-dispersive X-ray spectroscopy (EDXS). Scanning electron microscopy (SEM) was used for the microstructural and morphological analyses of specimens after compressive failure to elucidate the deformation mechanisms.

## Additional Information

**How to cite this article**: Dusoe, K. J. *et al*. Strong, ductile, and thermally stable Cu-based metal-intermetallic nanostructured composites. *Sci. Rep.* **7**, 40409; doi: 10.1038/srep40409 (2017).

**Publisher's note:** Springer Nature remains neutral with regard to jurisdictional claims in published maps and institutional affiliations.

## Supplementary Material

Supplementary Information

## Figures and Tables

**Table 1:** Comparison with other rapidly solidified MINCs.

MINC	σ_y_ (MPa)	σ_max_ (MPa)	ε_p_ (%)	Diameter (mm)	Ref.
Cu_90_Zr_7.5_Ti_2.5_ (This study)	1700	2110	18	1.5	—
Cu_90_Zr_7.5_Ti_2.5_ (This study)	1520	1630	11	3	—
Cu_85_Zr_10_Ti_5_ (This study)	1750	1910	9.5	1.5	—
Cu_85_Zr_10_Ti_5_ (This study)	1500	1590	3	2	—
Cu_85_Zr_10_Ti_5_ (This study)	1380	1510	2	3	
Cu_85_Zr_10_Ti_5_ (This study)	1200	1250	1.5	ingot	
Cu_85_Zr_10_Al_5_ (This study)	1800	2160	13	1.5	—
Ti_60_Cu_14_Ni_12_Sn_4_Nb_10_	1312	2401	14.5	3	28
Ti_60_Cu_14_Ni_12_Sn_4_Ta_10_	1568	2322	6.0	3	28
Ti_50_Cu_23_Ni_20_Sn_7_	1300	2114	5.5	3	28
Fe_74_Nb_11_Ni_10_Mn_5_	1400	1600	10	2	29

The yield strength of our Cu-based MINCs is superior to those of Ti-, and Fe-based MINCs. Competing materials are of similar specimen size, therefore having undergone similar cooling rates, yet they are composed of more alloying elements than the Cu-based MINCs presented in this work.
