# Supplementary material for: Strong, ductile, and thermally stable Cu-based metal-intermetallic nanostructured composites
Source: Sci Rep. 2017 Jan 9;7:40409. doi: 10.1038/srep40409 (PMC5220353; doi:10.1038/srep40409)
Supplement: Supplementary Information [file srep40409-s1.pdf]

Supplementary Information

Title: Strong, ductile, and thermally stable Cu-based metal-intermetallic nanostructured composites

Authors:

Keith J. Dusoe, Sriram Vijayan, Thomas R. Bissell, Jie Chen, Jack E. Morley, Leopolodo Valencia Jr., Avinash M. Dongare, Mark Aindow, Seok-Woo Lee*

Department of Materials Science and Engineering & Institute of Materials Science, 97 North Eagleville Road, Unit 3136, Storrs, CT, 06269-3136, USA

Corresponding Author:

Seok-Woo Lee

- **Address)** Department of Materials Science and Engineering & Institute of Materials Science, 97 North Eagleville Road, Unit 3136 Storrs, CT 06269-3136, USA
- **Email)** seok-woo.lee@uconn.edu
- **Telephone)** 860-486-8028

1. Cu-Zr Binary Phase diagrams

There are three major Cu-Zr binary phase diagrams reported to date. Phase formation mechanisms in Cu-rich sections (>90 at.%) in phase diagrams are still under debate. Different thermal treatments produce Cu_5Zr, $Cu_{51}Zr_{14}$, and Cu_9Zr in this composition region. The crystal structure of Cu_5Zr features Cu and Zr alternating layers, which produces the superlattice diffraction pattern in Figure 1 of the manuscript. However, rapid solidification produces chemical inhomogeneity and internal strain, making analysis of the diffraction data very difficult.

(1) **Ref. 20 of the manuscript**: Cu_5Zr is stable phase at room temperature.

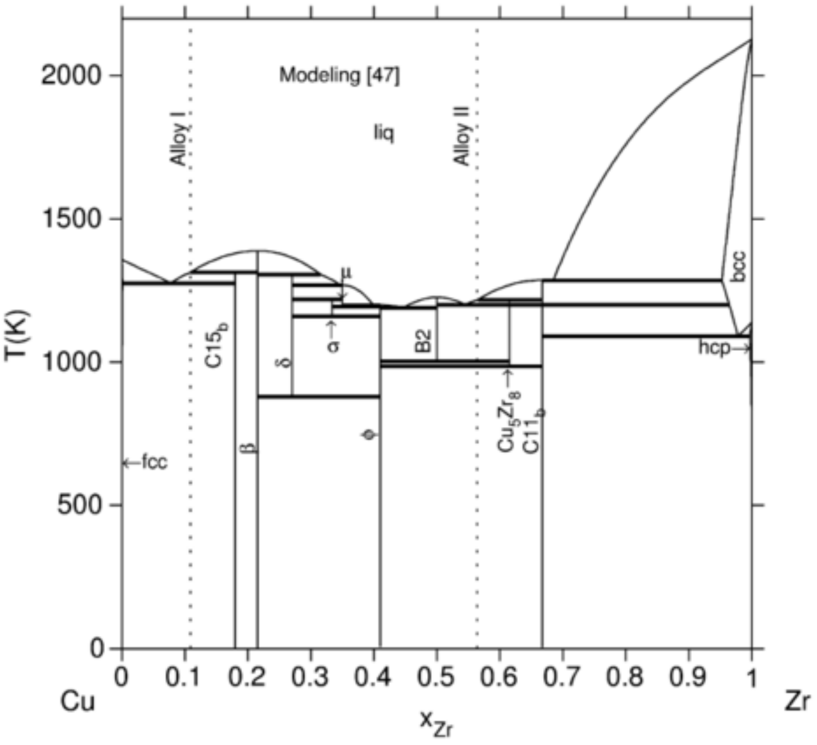

Figure S1. Cu-Zr phase diagram.

(2) **Ref. 21 of the manuscript**: Cu_9Zr_2 is the stable phase at room temperature.

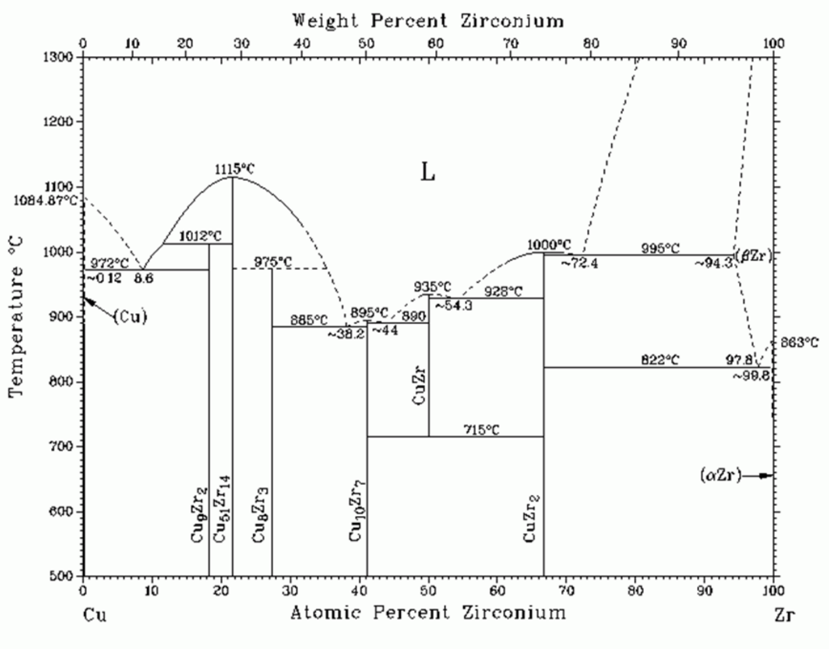

Figure S2. Cu-Zr phase diagram.

(3) **Ref. 22 of the manuscript**: $Cu_{51}Zr_{14}$ is the stable phase, and Cu_5Zr is the metastable phase at room temperature.

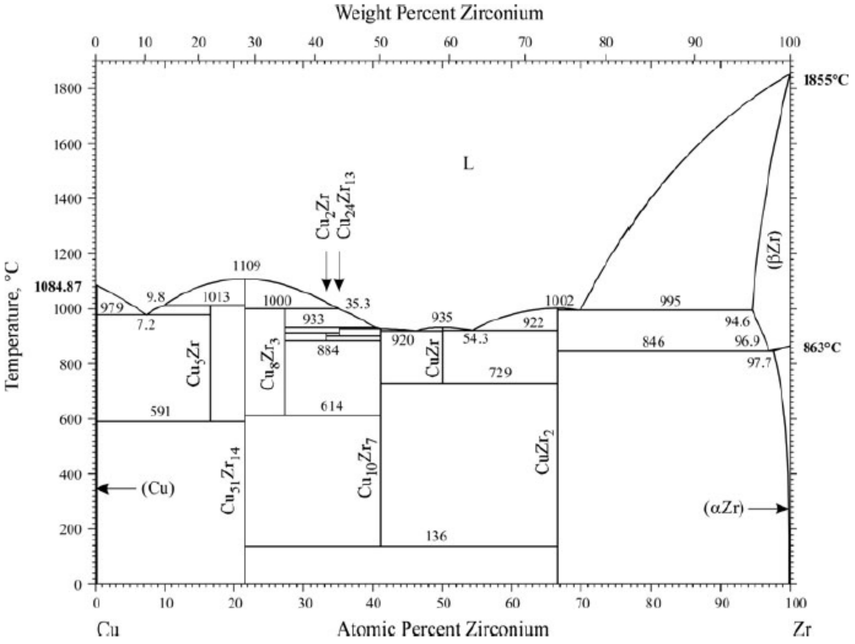

Figure S3. Cu-Zr phase diagram.

2. X-ray diffraction data and additional TEM analysis.

We performed X-ray diffraction (XRD) analysis for Cu-Zr-Al and Cu-Zr-Ti systems as well as TEM analysis on Cu-Zr-Ti system. For Cu-Zr-Ti system, XRD results showed Cu, Cu_5Zr, and Cu_4Ti phases (Figure S4(a)). Note that Cu_4Ti was not shown in our previous manuscript. In order to confirm the existence of Cu_4Ti phase, we performed additional TEM analysis. The bright field image and diffraction pattern with the [1 0 -1] zone axis confirms that Cu_4Ti phase indeed exists. The composition map in HAADF image also confirms that Cu_4Ti phase does not include Zr (Figure S5). However, we noticed that the volume fraction of Cu_4Ti is much smaller than that of Cu and Cu_5Zr. For Cu-Zr-Al system, XRD results show Cu, Cu_5Zr, and nanocrystalline phases (Figure S4(b)). These results are consistent with our TEM results in Figure 1. Also, there is a peak that the database could not index. As discussed in the first paragraph of Results section (page 4 of the manuscript), the phase formation mechanism in Cu-Zr binary system is still under debate, and several different phases seem to be formed within the narrow range of composition. Also, rapid solidification process often causes severe lattice distortion. Thus, minor peaks would be associated with unknown phases and severe lattice distortion, so it is difficult to index them. In conclusion, Cu and Cu_5Zr phases occupy the major volume fraction of Cu-based MINCs although there are minor phases such as nanocrystalline phase in Cu-Zr-Al system and Cu_4Ti phase in Cu-Zr-Ti system. Thus, Cu and Cu_5Zr phases would primarily control mechanical properties of our MINCs.

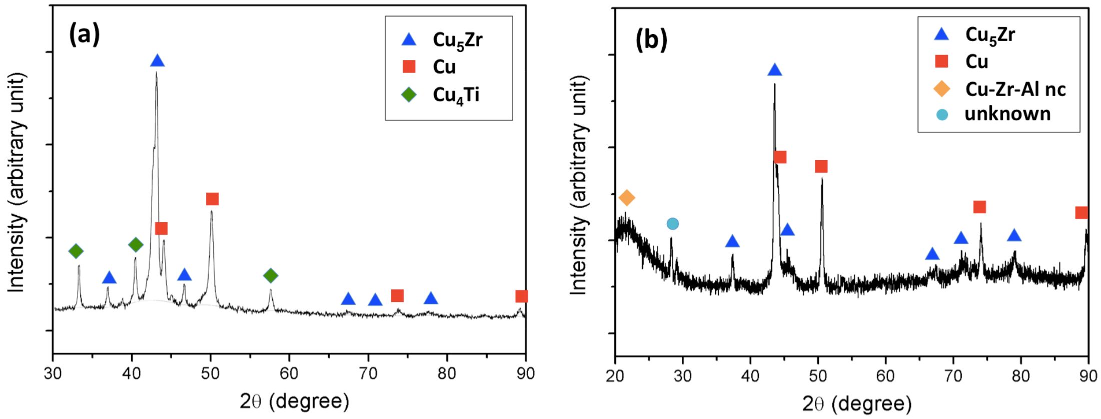

Figure S4. X-ray diffraction data of (a) $Cu_{85}Zr_{10}Ti_5$ and (b) $Cu_{85}Zr_{10}Al_5$.

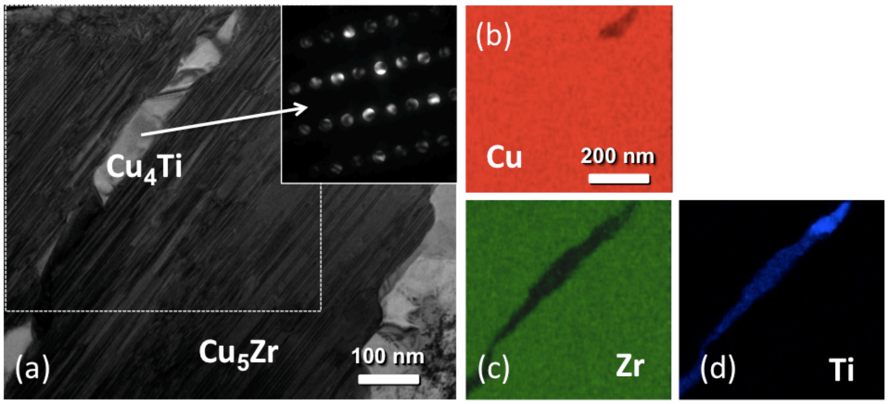

Figure S5. (a) The TEM bright field image of Cu_4Ti and Cu_5Zr phases in $Cu_{85}Zr_{10}Ti_5$ and HAADF composition map of (b) Cu, (c) Zr, and (d) Ti at the area of broken line box in Figure S4(a).

3. High angular annular dark field (HADDF) analysis

Ti has good solid solubility in Cu at an elevated temperature (7.5 at.% at 900 ^{o}C), but negligible solubility at room temperature. By rapidly solidifying our alloy, the diffusion of Ti atoms from Cu grains, which would typically occur with conservative cooling, is halted, creating a non-equilibrium Cu phase which is supersaturated with Ti. Figure S6 shows that Ti atoms exist **only** in Cu grains. We were not able to find any Cu-Ti intermetallic compounds from both HADDF analysis and diffraction patterns. Thus, Ti atoms would exist as a solute atom in Cu grains.

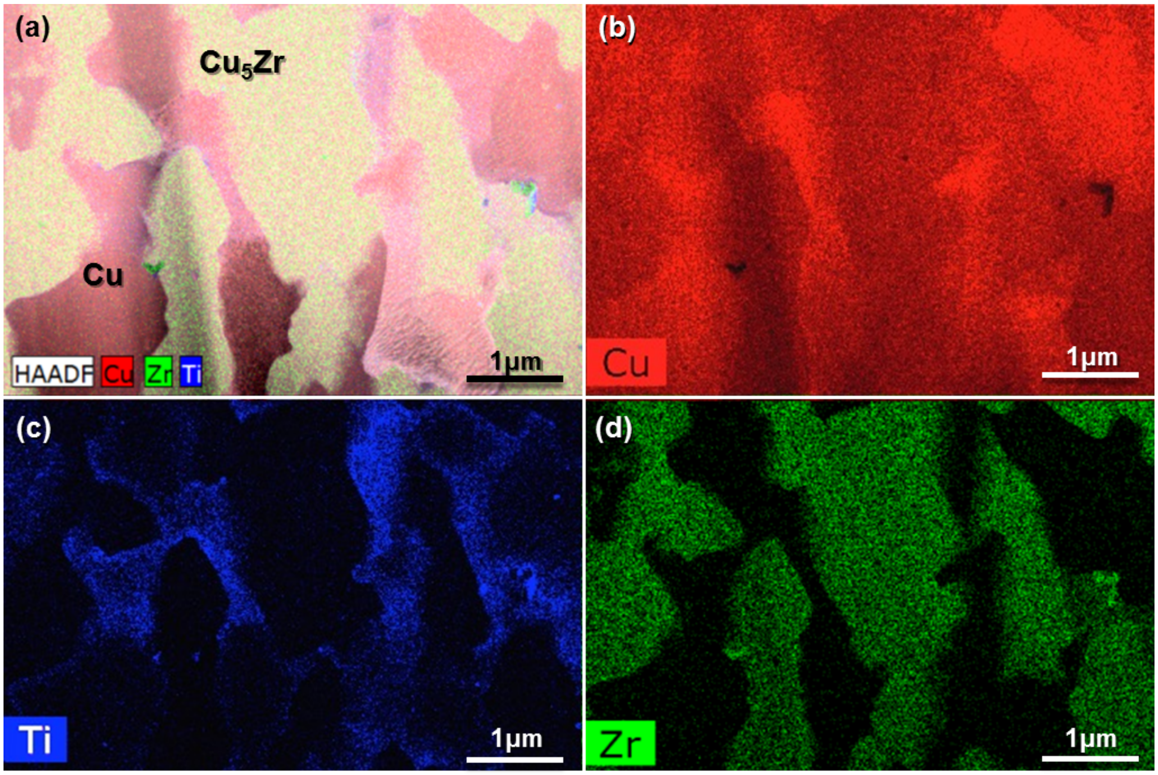

Figure S6. HADDF composition map of Cu, Ti and Zr in $Cu_{85}Zr_{10}Ti_5$ alloy. All Ti atoms exist only in Cu phase. Diffraction pattern does not show any Cu-Ti intermetallic compounds, which indicate that Cu and Ti forms supersaturated solid solution.

4. Reference information of Figure 2(b)

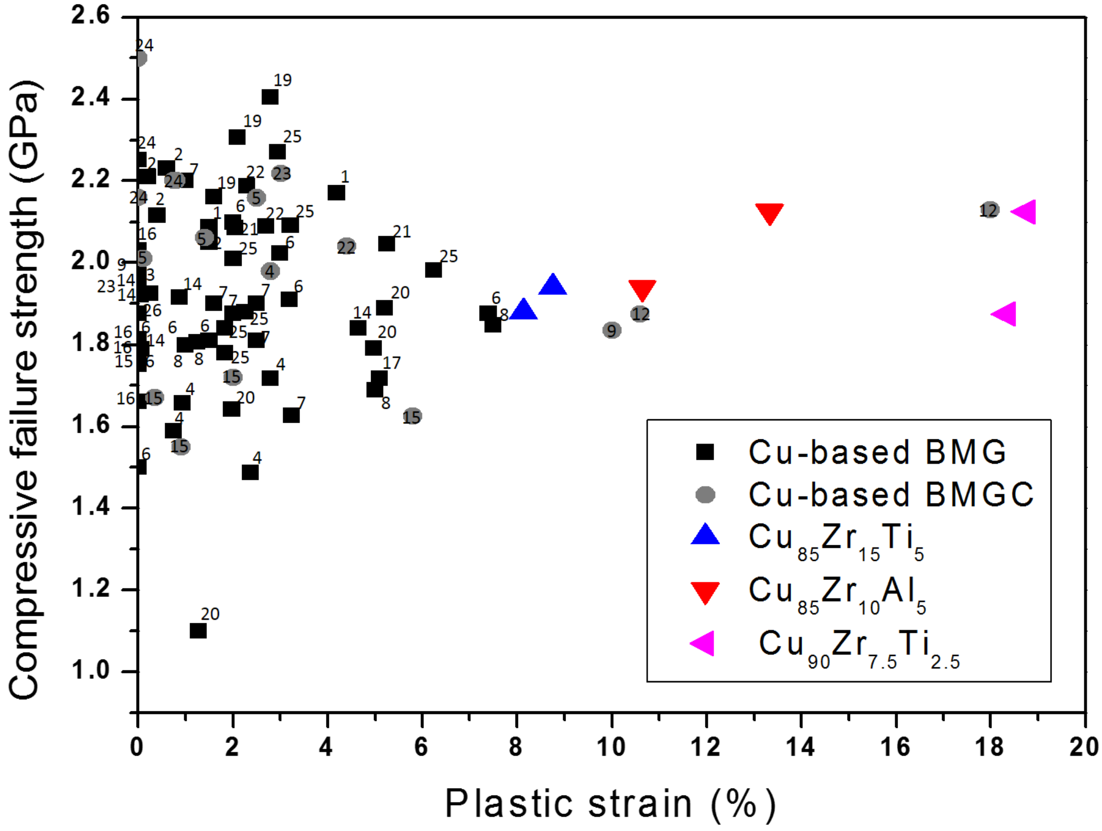

Figure S7. Plastic strain vs. compressive failure strength of Cu-based bulk metallic glassses (BMGs), Cu-based bulk metallic glass composites (BMGCs), and Cu-based metal-intermetallic nanostructured composites in this study. *Note that we included Cu-based materials that have Cu as a major element (the largest atomic percent) to consider Cu alloys only.*

The list of references is given below. The reference number below corresponds to the number in Figure S5.

1. Park ES, Kim DH, Ohkubo T, Hono K (2005) Enhancement of glass forming ability and plasticity by addition of Nb in Cu-Ti-Zr-Ni-Si bulk metallic glasses. *J Non Cryst Solids* 351(14-15):1231-1238

2. Zhang W, Qin C, Zhang X, Inoue A (2007) Effects of additional noble elements on the thermal stability and mechanical properties of Cu-Zr-Al bulk glassy alloys *Mat Sci Eng A* 449-451: 631-635

3.Concustell A, Alcala G, Mato S, Woodcock TG, Gebert A, Eckert J, Baro MD (2005) Effect of relaxation and primary nanocrystallization on the mechanical properties of $Cu_{60}Zr_{22}Ti_{18}$ bulk metallic glass. *Intermetallics* 13(11): 1214-1219

4. Abbasi M, Gholamipour R, Shahri F (2013) Glass forming ability and mechanical properties of Nb-containing Cu-Zr-Al based bulk metallic glasses *Transactions of Nonferrous Metals Society of China* 23(7): 2037-2041

5. Liu T, Shen P, Qiu F, Yin Z, Lin Q, Jiang Q, Zhang T (2009) Synthesis and mechanical properties of TiC-reinforced Cu-based bulk metallic glass composites *Scr Mater* 60(2):84-87

6 Pan Y, Zeng Y, Jin L, Zhang L, Pi J (2014) Composition design and mechanical properties of ternary Cu-Zr-Ti bulk metallic glasses. *Mater Des* 55:773-777

7. Kwon O, Lee Y, Park S, Lee J, Kim Y, Fleury E (2007) Thermal and mechanical behaviors of Cu-Zr amorphous alloys *Mat Sci Eng A* 449-451:169-171

8. Zhang T, Men H, Pang SJ, Fu JY, Ma CL, Inoue A (2007) Effect of a minor addition of Si and/or Sn on formation and mechanical properties of Cu-Zr-Ti bulk metallic glass. *Mat Sci Eng A* 449-451: 295-298

9. Malekan M, Shabestari SG, Zhang W, Seyedien SH, Gholaminpour R, Yubuta K, Makin A, Inoue A (2012) Formation of bulk metallic glass in situ nanocomposite in $(Cu_{50}Zr_{43}A_{l7})_{99}Si_{1}$ alloy *Mat Sci Eng A*553:10-13

10. Ma GZ, Sun BA, Pauly S, Song KK, Kuhn U, Chen D, Eckert J (2013) Effect of Ti substitution on glass-forming ability and mechanical properties of a brittle Cu-Zr-Al bulk metallic glass. *Mat Sci Eng A*563:112-116

11. Zhang W, Zhang Q, Qin C, Inoue A (2008) Synthesis and properties of Cu-Zr-Ag-Al glassy alloys with high glass-forming ability. *Mat Sci Eng B* 148(1-3):92-96

12. Zhang QS, Zhang W, Xie GQ, Nakayama KS, Kimura H, Inoue A (2007) Formation of bulk metallic glass in situ composites in $Cu_{50}Zr_{45}Ti_5$ alloy. *J Alloys Compd* 431(1-2):236-240

13. Baser TA, Eckert JD, Baricco M (2009) Glass formation and mechanical properties of $(Cu_{50}Zr_{50})_{100-x}Al_x$ (x=0,4,5,7) bulk metallic glasses. *J Alloys Compd* 483(1-2):146-149

14. Deng L, Zhoue B, Yang H, Jian X, Jiang B, Zhang X (2015) Roles of minor rare-earth elements addition in formation and properties of Cu-Zr-Al bulk metallic glasses. *J Alloys Compd* 632:429-434

15. Song KK, Pauly S, Sun BA, Zhang Y, Tan J, Kuhn U, Stoica M, Eckert J (2012) Formation of Cu-Zr-Al-Er bulk metallic glass composites with enhanced deformability. *Intermetallics* 30:132-138

16. Pi J, Pan Y, Wu J, Zhang L, He X (2013) Preparation and properties of novel Cu-based bulk metallic glasses $Cu_{55-x}Zr_{37}Ti_8In_x$. *Transactions of Nonferrous Metals Society of China* 23(10):2989-2993

17. Wang G, Pauly S, Gorantla S, Mattern N, Eckert J (2014) Plastic Flow of a $Cu_{50}Zr_{45}Ti_5$ Bulk Metallic Glass Composite *Journal of Materials Science & Technology* 30(6):609-615

19. Qin C, Zhang W, Asami K, Ohtsu N, Inoue A (2005) Glass formation, corrosion behavior and mechanical properties of bulk glassy Cu-Hf-Ti-Nb alloys *Act Mater* 53(14): 3903-3911

20. Park ES, Kim DH (2006) Phase separation and enhancement of plasticity in Cu-Zr-Al-Y bulk metallic glasses *Act Mater* 54(10):2597-2604

21. Park ES, Change HJ, Kim DH (2008) Effect of addition of Be on glass-forming ability, plasticity and structural change in Cu-Zr bulk metallic glasses. *Act Mater* 56(13):3120-3131

22. Calin M, Eckert J, Schultz L (2003) Improved mechanical behavior of Cu-Ti-based bulk metallic glass by in situ formation of nanoscale precipitates. *Scr Mater* 48(6):653-658

23. Kato H, Yubuta K, Louzguine DV, Inoue A, Kim HS (2004) Influence of nanoprecipitation on strength of $Cu_{60}Zr_{30}Ti_{10}$ glass containing um_ZrC particle reinforcements. *Scr Mater* 51(6): 577-581

24. Fu H, Zhang H, Wang H, Zhang Q, Hu Z (2005) Synthesis and mechanical properties of Cu-based bulk metallic glass composites containing in-sit TiC particles. *Scr Mater* 52(7):669-673

25. Park ES, Chang HJ, Kim DH, Ohkubo T, Hono K (2006) Effect of the substation on Ag and Ni for Cu on the glass forming ability and plasticity of $Cu_{60}Zr_{30}Ti_{10}$ alloy. *Scr Mater* 54(9):1569-1573

26. Zhang Q, Zhang W, Inoue A (2006) New Cu-Zr-based bulk metallic glasses with large diameters of up to 1.5cm. *Scr Mater* 55(8):711-713

5. Strong chemical resistance and large volume fraction of Cu-Zr intermetallic compound

After short exposure to a concentrated solution of Nital etchant (50% nitric acid, 50% methanol), preferential and complete chemical attack on the Cu phase (with Ti solutes) is observed. The persistence of the Cu-Zr intermetallic phase illustrates the resistance of this phase to chemical dissolution. Additionally, the distribution of the Cu-Zr intermetallic phase is highlighted in the absence of the Cu phase, illustrating its large volume fraction in our material. From TEM image analysis, the volume fraction of intermetallic phase is nearly 60 % (Note: The volume fraction measurement cannot be done with Figure S8 because Cu-Zr intermetallic phases are stacked up after removing Cu phases.).

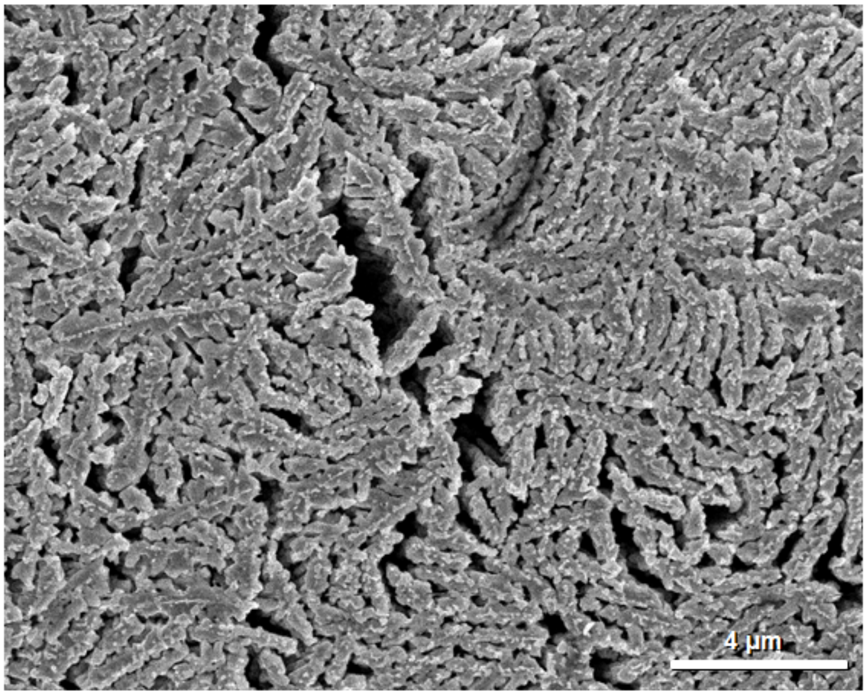

Figure S8. Scanning electron microscope image of $Cu_{85}Zr_{10}Ti_5$ after short exposure to a concentrated solution of Nital etchant (50% nitric acid, 50% methanol).

6. Theoretical shear strength of Cu_5Zr and Cu

First-principles density functional theory calculations are carried out using Vienna ab initio simulation package (VASP) to obtain the theoretical strength in the most favorable slip systems in Cu_5Zr phase [1, 2], with the electronic-ion interaction described by a projector augmented wave method [3, 4] and a Perdew-Burke-Ernzerhof form generalized gradient approximation (GGA) [5] for the exchange-correlation potential. A plane wave basis sets wave function is expanded with a cutoff energy of 400eV. Brillouin zone integration was performed using a Monkhorst-Pack scheme [6], with a k-point mesh of 4x4x1 for {001} slip systems (6.89x6.89x23.78 $Å^3$) and 6x3x1 for {111} slip systems (4.87x8.44x25.91 $Å^3$). The crystal structure of Cu_5Zr phase is shown in Figure S9.

Density functional theory (DFT) calculations were carried out to determine and compare the resistance to plastic slip in Cu_5Zr and Cu phase. The generalized stacking fault (GSF) energies were calculated for various slip planes and directions (Figure S10). We carefully made the list of the potential slip planes based on the interplanar spacing. Typically, the large interplanar spacing enables the easy slip process. We found that the minimum energy barrier for slip in Cu_5Zr (~1000 mJ/m^2) is much higher than that in Cu (~250 mJ/m^2), which suggests a significantly high slip resistance as well as high strength in the Cu_5Zr intermetallic compound phase. Also, GSF curves show that the theoretical shear strength of Cu_5Zr is close to 8 GPa, but that of Cu is ~3 GPa. Therefore, Cu_5Zr is much stronger than Cu, and the large volume fraction (~60 %) of intermetallic phase would make the major contribution to the high strength of our MINCs.

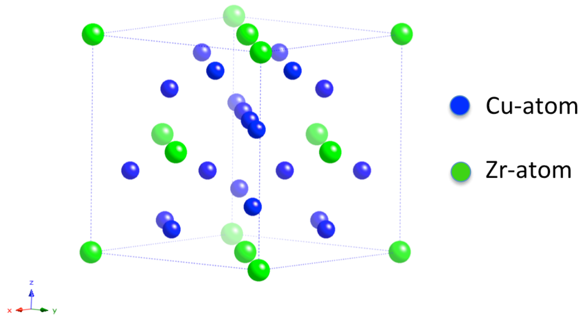

Figure S9. Unit cell of Cu_5Zr intermetallic compound phase (Space group: F-43m, a = 0.687 nm)

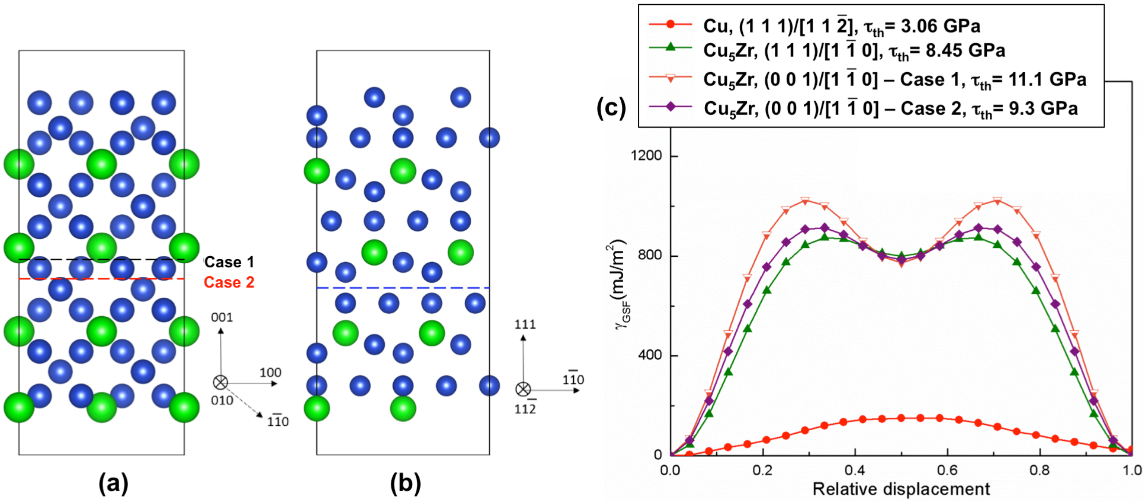

Figure S10. (a) Two (0 0 1)/[1 -1 0] slip systems and (b) one (1 1 1)/[1 -1 0] slip system of Cu_5Zr IM phase. These slip systems are chosen based on the largest interplanar spacing, which is the pre-requisite of preferred slip process. (c) Generalized stacking fault energy curves of Cu_5Zr and Cu phases.

References

1. G. Kresse and J. Furthmüller, *Comp. Mater. Sci.* **6**, 15 (1996).
2. G. Kresse and J. Furthmuller, *Phys. Rev. B*. **54**, 11169 (1996).
3. P. E. Blochl. *Phys. Rev. B*. **50**, 17953 (1994).
4. G. Kresse and D. Joubert, *Phys. Rev. B*. **59**, 1758 (1999).
5. J. P. Perdew, K. Burke, and M. Ernzerhof, *Phys. Rev. Lett*. **77**, 3865 (1996).
6. H. J. Monkhorst and J. D. Pack, *Phys. Rev. B*. **13**, 5188 (1976).

7. The effects of cooling rate on ductility of $Cu_{85}Zr_{10}Ti_5$

Cylindrical specimens having diameters of 1.5, 2 and 3mm were tested in compression to illustrate the effects of cooling rate on the ductility. Additionally, 2x2x4mm rectangular specimens were machined from the bulk pre-alloying ingot and compressed. Obviously, the bulk ingot undergoes solidification at the slowest cooling rate and the 1.5mm diameter casting is solidified at the highest rate observed in this work. A simultaneous increase in ductility and strength is observed as specimens of similar composition, $Cu_{85}Zr_{10}Ti_5$, are solidified with an increasing rate of cooling (Table 1 and Figure S11).

Based on the observation in Figure 3, ductility in Cu-based MINCs is not due to conventional dislocation-based plastic deformation mechanisms. Rather, it is the result of shear displacement at phase boundaries between metallic and intermetallic phases. In D~1.5 mm specimen, the ultra-fine microstructure of our rapidly cooled specimens would provide an optimum morphology and connectivity of phase boundaries. The elongated shape of the intermetallic phases, which are oriented 45 degrees from the loading direction, serves as a smooth sliding surface which allows for shear-driven phase boundary sliding to occur easily and accounts for the extensive compressive plasticity (~20%) observed. Intermetallic phases, which are not oriented along 45 degrees, would effectively obstruct the continuous propagation of shear deformation, preventing catastrophic brittle fracture from occurring. However, D~3 mm or ingot specimen contains coarser intermetallic compounds and more rough connectivity of phase boundary, leading to the formation of large cracks in intermetallic compounds and preventing effective phase boundary sliding, respectively. Figure S12 shows the formation of large cracks in coarse intermetallic compounds, which are not observed in D~1.5 mm specimen. These large cracks would propagate through a specimen, leading to early fracture.

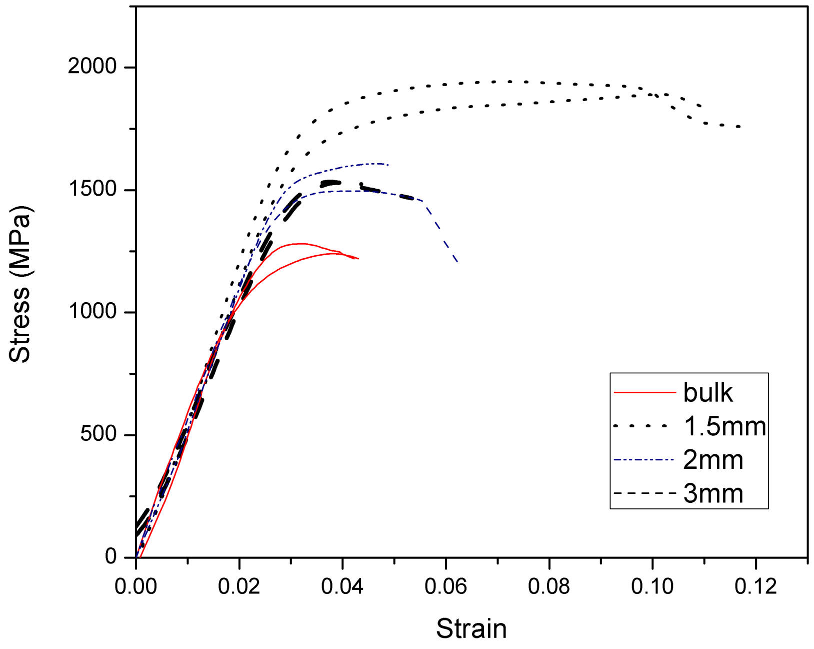

Figure S11: Stress-strain plot illustrating the cooling rate effect upon ductility for MINC having composition of $Cu_{85}Zr_{10}Ti_5$. Ductility in Cu based MINCs has a strong cooling rate effect.

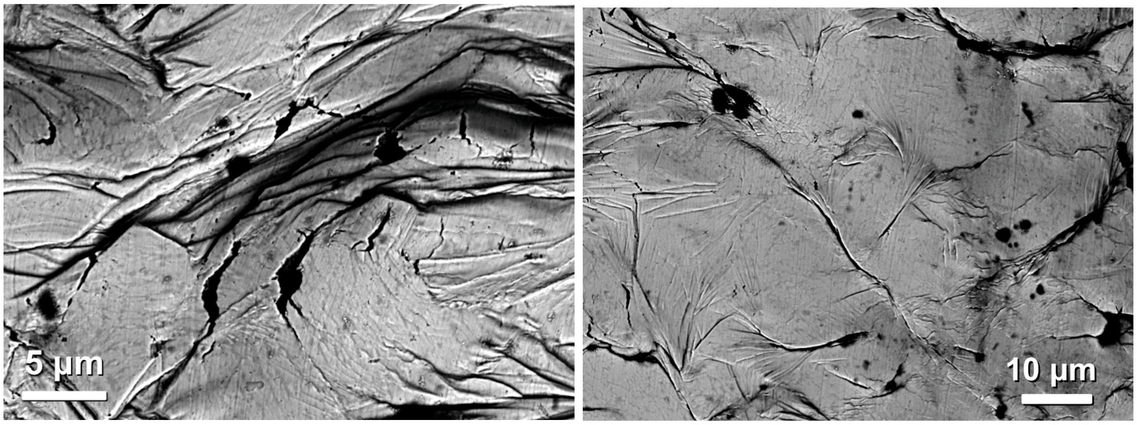

Figure S12. The development of large cracks in D~3mm $Cu_{85}Zr_{10}Ti_5$ specimen after compression test.
